# Comparison of anterior and posterior approach in the treatment of acute and chronic cervical spinal cord injury: a meta-analysis

**DOI:** 10.3389/fsurg.2024.1410220

**Published:** 2024-08-23

**Authors:** Yi Ding, Ning Li, Wenjing Hu, Wenkang Jiang, Qianmiao Zhu, Ting Jiang, Huilin Cheng

**Affiliations:** ^1^ School of Medicine, Southeast University, Nanjing, China; ^2^ Department of Neurosurgery, Zhongda Hospital, Southeast University, Nanjing, China; ^3^ School of Public Health, Southeast University, Nanjing, China

**Keywords:** cervical spinal cord injury, outcomes, anterior approach, posterior approach, meta-analysis

## Abstract

**Objective:**

A cervical spinal cord injury (CSCI) is a traumatic catastrophe that often leads to neurological dysfunction. The optimal surgical procedure for the treatment of CSCI remains debatable. The aim of this meta-analysis is to compare the neurological outcomes, complications, and clinical factors between anterior and posterior approach in CSCI treatment.

**Methods:**

We searched PubMed, Embase, Web of Science and Cochrane library from their inceptions to october 2023. Preoperative and postoperative Spinal Injury Association (ASIA) and Japanese Orthopedic Association (JOA) scores, and calculated recovery rates (RRs) were compared between the two strategies, and differences in complication rates, operation time, intraoperative blood loss and length of stay were also analyzed.

**Results:**

A total of five studies containing 613 patients were included, with 320 patients undergoing the anterior approach and 293 patients undergoing the posterior approach. Four of the studies included were retrospective cohort studies of high quality as assessed by the Newcastle Ottawa Scale. Additionally, there was one randomized controlled trial evaluated with the Cochrane Risk of Bias tool. Although both anterior and posterior approaches effectively facilitate spinal decompression and promote good neurological recovery, there was no significant difference in the incidences of neurological dysfunction and complications or other clinical features between the two approaches.

**Conclusion:**

There is no evidence thus far supports one approach over the other. Large-scale randomized controlled studies are warranted to further distinguish these two methods.

**Systematic Review Registration:**

https://www.crd.york.ac.uk/, PROSPERO [CRD42023438831].

## Introduction

Cervical spinal cord injury (CSCI) is a form of neurological trauma affecting the cervical spinal cord, often resulting in severe consequences such as sensorimotor impairment, paralysis, or even death. While traumatic events such as traffic accidents or falls are the predominant causes of CSCI, cervical degeneration and the progressive narrowing of the spinal canal due to multilevel cervical disc herniation or ossification of the posterior longitudinal ligament can also lead to chronic spinal cord injury or compression, clinically termed as degenerative cervical myelopathy (DCM) (1). It is important to note the different pathophysiological processes involved. In the setting of traumatic CSCI, the initial impact causes stretches and tears in the spinal cord, leading to a sudden impairment in neurological function. This primary injury is often followed by a series of secondary injuries, including inflammation, demyelination, and glial scar formation (2). Surgical decompression and immobilization are the primary treatment modalities for CSCI (3). Among various surgical techniques, the anterior and posterior approaches are most employed (4). While both have their merits and drawbacks, the optimal approach remains uncertain. This meta-analysis aims to compare the clinical outcomes of these two approaches in terms of neurological recovery, complications, and other clinical factors in the setting of traumatic CSCI.

## Materials and methods

### Study protocol

The study protocol is registered on the PROSPERO website under the registration number CRD42023438831.

### Search strategy

We performed a comprehensive search of PubMed, Embase, Web of Science, and the Cochrane Library from their inception to October 2023 following PRISMA guidelines (5). The search strategy included a combination of terms related to anterior and posterior approaches and CSCI: (1) anterior OR anterior cervical corpectomy and fusion OR ACCF; anterior cervical discectomy with fusion or ACDF; (2) posterior OR laminectomy OR LA OR laminoplasty OR LP; and (3) cervical spinal cord injury OR CSCI; and (1) and (2) and (3). The reference lists of all relevant retrieved articles and reviews were manually searched to identify additional studies that might have been missed. Two independent reviewers screened the titles and abstracts, and full-text articles were obtained for further evaluation.

### Inclusion and exclusion criteria

Studies were included based on the following criteria: (1) Study design: Randomized or non-randomized controlled studies; (2) Study population: Persons with a history of traumatic CSCI; (3) Interventions: Comparison of clinical outcomes between anterior and posterior approaches; (3) Primary outcomes: Preoperative and postoperative Spinal Injury Association (ASIA) and Japanese Orthopedic Association (JOA) scores, calculated recovery rates (RRs) (%); (4) Secondary outcomes: Complication rate, operation time, intraoperative blood loss, and length of stay. Exclusion criteria included duplicate reports, non-English studies, studies lacking a control group, studies only with abstract or with unavailable statistical data. Reviews, case reports, letters, comments, animal trials or cadaver studies were also excluded.

### Data extraction

Data were extracted independently by two authors, and discrepancies were resolved through discussion with a third author. The following information was extracted: (1) Basic information including title, first author, year of publication, and study design; (2) Sample characteristics such as sample size, sex, and age; and (3) Interventions and clinical data including surgical approach for each group, preoperative and postoperative ASIA and JOA scores, RRs calculated based on JOA score, complication rate, operation time, intraoperative blood loss and length of stay.

### Quality assessment

The quality of the included studies was independently evaluated. Newcastle Ottawa Scale was introduced for cohort studies, and the Cochrane Risk of Bias tool was applied for randomized controlled trials, respectively. The Newcastle‒Ottawa Scale includes 3 domains: quality of selection, comparability, exposure, and outcome of study participants. A maximum of 9 points was assigned to each study, including 4 for selection, 2 for comparability, and 3 for outcomes. A study with a final score >6 was regarded as high quality (6, 7). The Cochrane risk of bias tool covers six domains of bias: selection bias, performance bias, detection bias, attrition bias, reporting bias, and other bias11. Each of these factors was recorded as yes (“low” risk of bias), no (“high” risk) or unclear.

### Data analysis

Data were analyzed using Review Manager (RevMan, Version 5.0). We employed either the inverse-variance method or the Mantel-Haenszel test to calculate effect sizes and their corresponding 95% confidence intervals. Forest plots were generated for visual representation of the data. We adopted a fixed-effects model to assess the overall impact of each approach on the primary and secondary outcomes. Statistical heterogeneity among the included studies was evaluated using the Cochrane Q test and quantified by the *I*^2^ statistic. In cases where the *p* value was less than 0.05 or the *I*^2^ statistic exceeded 50%, indicating significant heterogeneity, a random-effects model was employed for meta-analysis. Otherwise, a fixed-effects model was utilized. A *p* value of less than 0.05 was considered to indicate statistical significance (8).

## Results

### Search results and the quality assessment

**Figure 1:**
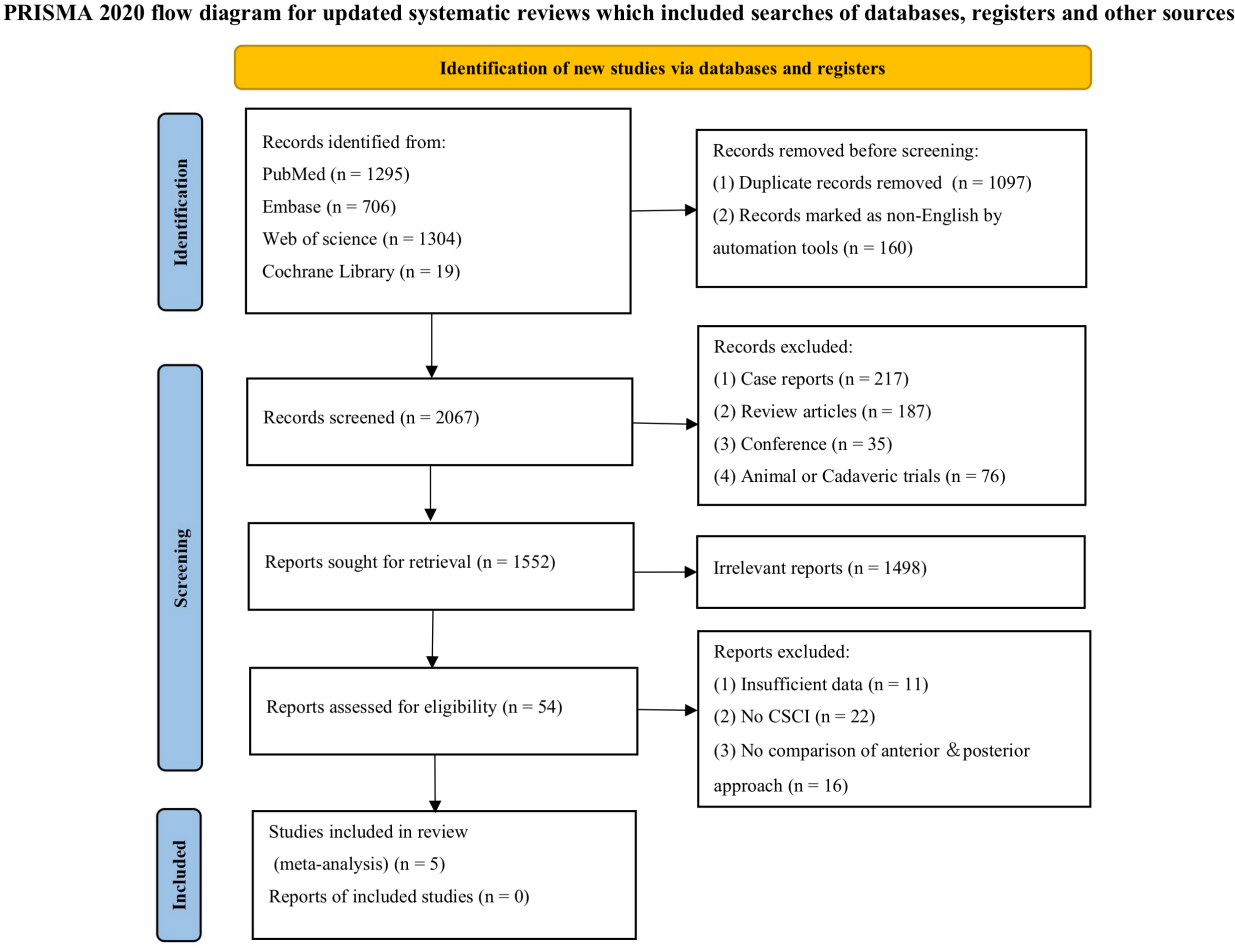
PRISMA flow diagram of the study selection process.

**Figure 2:**
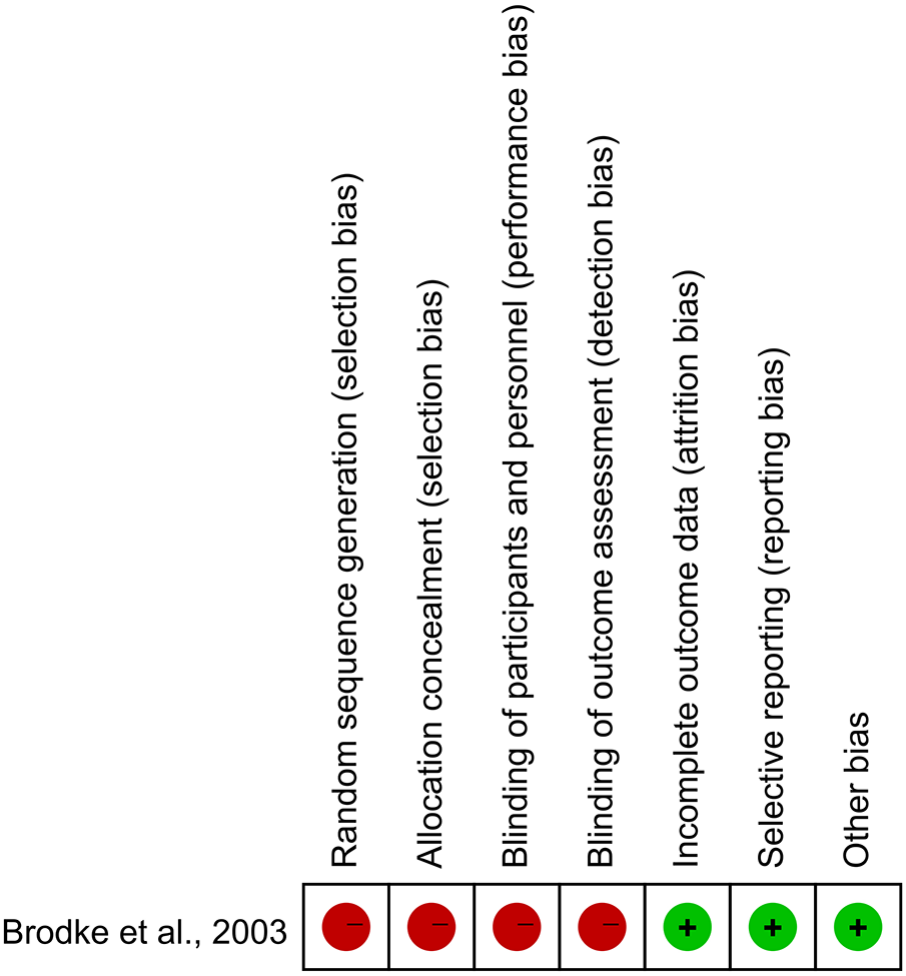
Quality assessment of the included studies according to the cochrane, and red represents high risk while green represents low risk.

Our search yielded 1,433 studies, of which five met the inclusion criteria after screening (Figure 1) (9–13). All five studies were conducted in Asia, with publication years ranging from 2003 to 2023. The sample sizes varied from 47 to 180, totaling 613 persons (320 with the anterior group and 293 with the posterior group, respectively). The follow-up length ranges from 6 months to 17 years (Table 1). The quality of the randomized control study (9) was evaluated by the Cochrane risk of bias tool (Figure 2), and quality of the included cohort studies (10–13) was evaluated using the Newcastle‒Ottawa scale (Table 2).

**Table 1:** Characteristics of the included studies.

Study	Design	The years of publication	Sample size (*N*)		Sex (M)		Patient age (Year)		Baseline ASIA score (Mean ± sd)		Baseline JOA score (Mean ± sd)		Follow-up (Mean)	
			A	P	A	P	A	P	A	P	A	P	A	P
Brodke et al. 2003 (9)	RCT	NA	20	27	15	22	38	33	2.15 ± 1.31	2.22 ± 1.50	NA	NA	>6 months	>6 months
Jia et al. 2023 (10)	Retrospective cohort study	2012–2019	84	84	56	53	33.24 ± 4.71	32.19 ± 3.95	2.56 ± 0.95	2.54 ± 0.93	7.10 ± 1.70	6.80 ± 2.10	NA	NA
Ren et al. 2020 (11)		2002–2009	92	67	63	44	53.10 ± 14.20	54.70 ± 15.60	3.10 ± 1.10	3.20 ± 1.10	9.50 ± 3.60	9.60 ± 3.40	13.40 years	12.70 years
Yin et al. 2022 (12)		2019–2021	89	91	53	54	41.30 ± 6.30	42.20 ± 7.60	2.45 ± 0.24	2.51 ± 0.31	8.89 ± 1.03	9.05 ± 1.25	NA	NA
Zhou et al. 2022 (13)		2015–2018	35	24	24	14	56.11 ± 9.29	59.46 ± 10.14	3.60 ± 0.60	3.29 ± 0.81	9.40 ± 2.37	8.63 ± 2.58	41.49 months	41.49 months

**Table 2:** Quality assessment of the included studies according to the Newcastle‒Ottawa scale.

Study	Selection	Comparability	Exposure	Total score
Jia et al. (10)	4	2	2	8
Ren et al. (11)	4	2	2	8
Yin et al. (12)	4	1	2	7
Zhou et al. (13)	4	1	3	8

### ASIA and JOA scores

**Figure 3:**
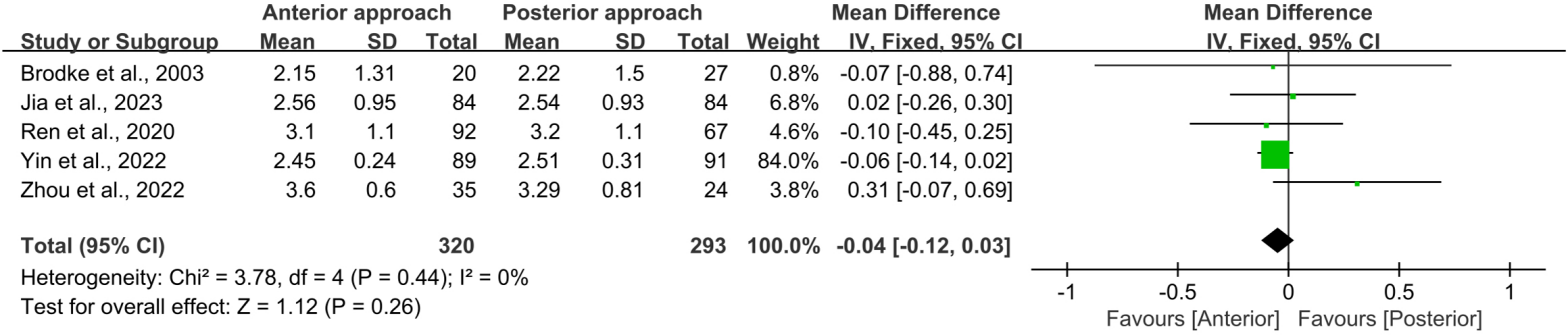
Forest plot of preoperative ASIA scores between the anterior surgery group and the posterior surgery group. CI, confidence interval; IV, inverse variance; SD, standard deviation.

**Figure 4:**
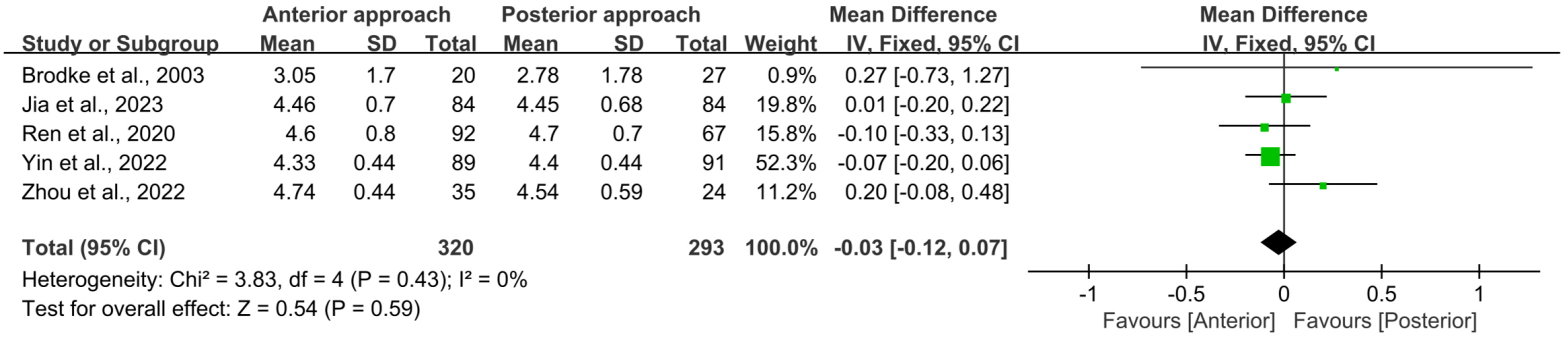
Forest plot of the difference in the postoperative ASIA score between the anterior surgery group and the posterior group at the postoperative follow-up. CI, confidence interval; IV, inverse variance; SD, standard deviation.

All five studies included in this meta-analysis reported both initial ASIA scores at admission (preoperative) and final ASIA scores at the end of the follow-up period (postoperative). Statistical analysis using the Chi-square test revealed no significant heterogeneity for either preoperative or postoperative ASIA scores (*I*^2^ = 0%, *p* = 0.44 and *I*^2^ = 0%, *p* = 0.43, respectively). Furthermore, no significant differences were observed between the anterior and posterior surgical approaches in terms of preoperative (WMD = −0.04, 95% CI −0.12 to 0.03, *p* = 0.26; Figure 3) or postoperative (WMD = −0.03, 95% CI −0.12 to 0.07, *p* = 0.59; Figure 4) ASIA scores.

**Figure 5:**
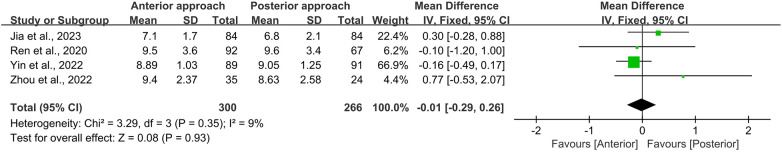
Forest plot of the difference in the preoperative JOA scores between the anterior surgery group and the posterior group. CI, confidence interval; IV, inverse variance; SD, standard deviation.

**Figure 6:**
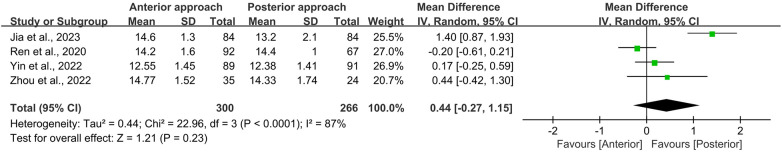
Forest plot of the difference in postoperative JOA scores between the anterior surgery group and the posterior group at the follow-up. CI, confidence interval; IV, inverse variance; SD, standard deviation.

Similarly, the initial JOA scores for persons undergoing either anterior or posterior surgical approaches were comparable (WMD = −0.01, 95% CI −0.29 to 0.26, *p* = 0.93; Figure 5). Postoperative JOA scores also showed no significant difference between the two groups (WMD = 0.44, 95% CI −0.27 to 1.15, *p* = 0.23; Figure 6). It is noteworthy that while the preoperative JOA data exhibited low heterogeneity (*I*^2^ = 9%, *p* = 0.35), the postoperative JOA data demonstrated high heterogeneity (*I*^2^ = 87%, *p* < 0.0001).

### Recovery rate

RR was quantified using the formula:

$$\mathrm{RR}(\text{\%})=\frac{\mathrm{JOA}\;\mathrm{score}\;\mathrm{at}\;\mathrm{last}\;\mathrm{follow}\text{-}\mathrm{up}\;\text{-}\;\mathrm{Preoperative}\;\mathrm{JOA}\;\mathrm{score}}{17\;\text{-}\;\mathrm{Preoperative}\;\mathrm{JOA}\;\mathrm{score}}\times 100$$

**Figure 7:**
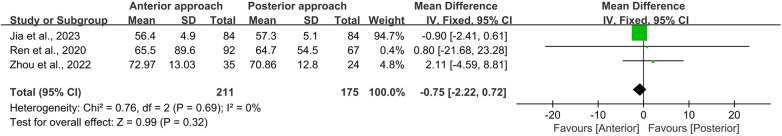
Forest plot of the difference in recovery rate between the anterior surgery group and the posterior group. CI, confidence interval; IV, inverse variance; SD, standard deviation.

This metric was employed in three of the studies to gauge the extent of neurological functional improvement (10, 11, 13). The meta-analysis revealed no significant difference in neurological RR between the anterior and posterior surgical approaches (WMD = −0.75, 95% CI −2.22 to 0.72, *p* = 0.32; Figure 7). Additionally, the Cochrane Q test indicated an absence of heterogeneity among these studies (*I*^2^ = 0%, *p* = 0.69).

### Complications

In the present meta-analysis, a cohort of 445 persons was evaluated across four studies (9, 11–13). Of these, 61 persons (25.8%) who were participated in the anterior surgical approach and 50 persons (23.9%) who underwent the posterior approach reported postoperative complications. The predominant complications encompassed odynophagia, hoarseness, neck pain, and wound infection, as delineated in Table 3.

**Table 3:** Number of complications (percentage of included patients).

Complication	Anterior (*N* = 61)	Posterior (*N* = 50)
Deep vein thrombosis	1 (1.9%)	NA
Adjacent segment degeneration	1 (1.9%)	NA
Odynophagia	19 (36.5%)	1 (2.5%)
Hoarseness	13 (25.0%)	10 (25.0%)
Dysphagia	4 (6.6%)	NA
Neck pain	10 (16.4%)	18 (36%)
Internal fixation loosening	5 (8.2%)	4 (8.0%)
Infected wound	3 (5.8%)	7 (17.5%)
Lung infection	3 (4.9%)	4 (8.0%)
Esophageal injury	2 (3.8%)	5 (12.5%)
C5 nerve root paralysis	NA	1 (2.5%)

**Figure 8:**
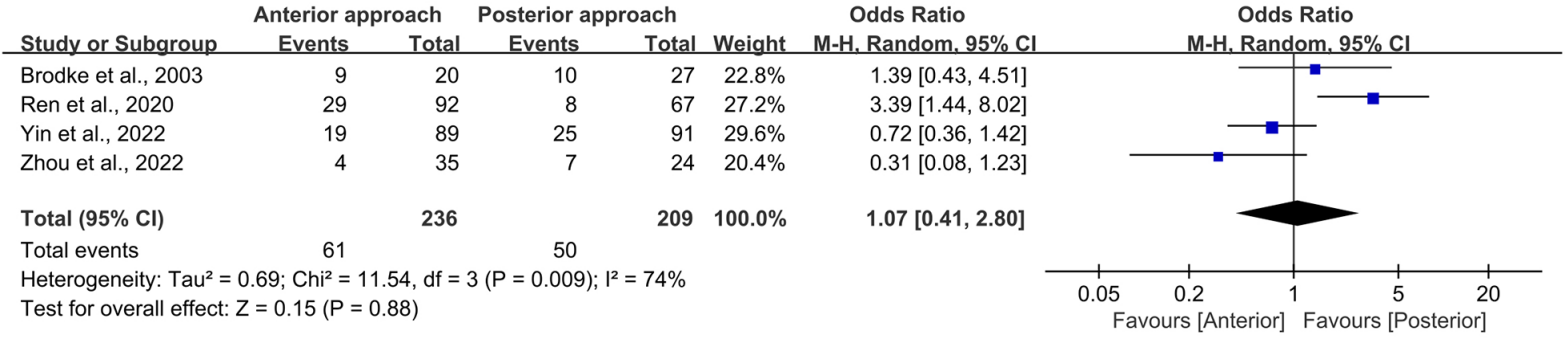
Forest plot of the difference in complication rate between the anterior surgery group and the posterior group. CI, confidence interval; M–H, Mantel–Haenszel.

Upon statistical scrutiny, no discernible difference was observed in the frequency of complications between the anterior and posterior surgical approaches (OR = 1.07, 95% CI 0.41–2.80, *p* = 0.88; Figure 8). It is noteworthy that a significant level of heterogeneity was detected among the included studies (*I*_2_ = 74%, *p* = 0.009).

**Figure 9:**
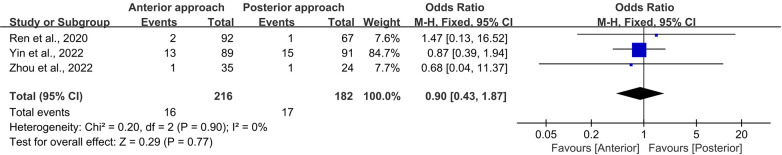
Forest plot of the difference in complication rate (infected wound and hoarseness) between the anterior surgery group and the posterior group. CI, confidence interval; M–H, Mantel–Haenszel.

To further validate these findings, a sensitivity analysis was executed, focusing on universally reported complications such as infected wounds and hoarseness. This analysis corroborated the stability of our primary results, revealing no heterogeneity (*I*^2^ = 0%, *p* = 0.90) and an OR of 0.90 (95% CI 0.43–1.87, *p* = 0.77; Figure 9).

### Operation time and intraoperative blood loss

**Figure 10:**
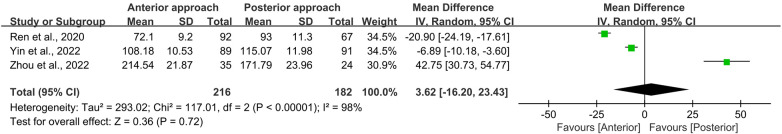
Forest plot of the difference in operation time between the anterior surgery group and the posterior group. CI, confidence interval; IV, inverse variance; SD, standard deviation.

**Figure 11:**
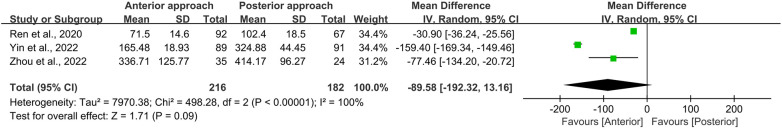
Forest plot of the difference in intraoperative blood loss between the anterior surgery group and the posterior group. CI, confidence interval; IV, inverse variance; SD, standard deviation.

In this meta-analysis, operation time and intraoperative blood loss were evaluated across three studies, encompassing a total of 398 persons—216 treated with the anterior approach and 182 with the posterior approach (11–13). Statistical analysis revealed no significant disparities in either operation time (*p* = 0.72; Figure 10) or intraoperative blood loss (*p* = 0.09; Figure 11) between the two surgical approaches. However, it is imperative to note the presence of substantial heterogeneity in these datasets (*I*^2^ = 98% for operation time, and 100% for intraoperative blood loss). This heterogeneity is postulated to emanate from variations in the specific surgical techniques employed, precluding the possibility of subgroup analyses due to the limited scope of the included literature.

### Length of stay

**Figure 12:**
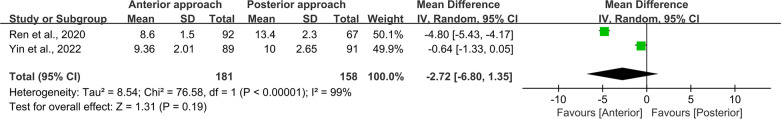
Forest plot of the difference in length of stay between the anterior surgery group and the posterior group. CI, confidence interval; IV, inverse variance; SD, standard deviation.

Two studies endeavored to elucidate the differences in hospital stay duration between the anterior and posterior surgical approaches (11, 12). Statistical analysis yielded a high degree of similarity (WMD = −2.72, 95% CI −6.80 to 1.35, *p* = 0.19; Figure 12), albeit with pronounced heterogeneity (*I*^2^ = 99%, *p* < 0.0001). We posit that this heterogeneity is likely attributable to variations in hospital environments, as the studies sourced their samples from different settings, thereby introducing significant disparities.

## Discussion

Cervical Spinal Cord Injury (CSCI) is a debilitating condition affecting the central nervous system, often resulting in partial or complete loss of motor and/or sensory functions (14). The pathophysiology involves a cascade of events, including hemorrhage, edema, and dural adhesions, which elevate intradural pressure and trigger ischemic and hypoxic processes, exacerbating secondary injury to the spinal cord (15). Current treatment for CSCI encompass surgical decompression, immobilization, and pharmacotherapy. Although surgery cannot reverse the initial injury, it can stabilize the cervical spine and alleviate compression, thereby mitigating secondary injury. Our meta-analysis corroborates that surgical intervention significantly improves postoperative ASIA and FIM motor scores compared to nonoperative treatment (16).

While both anterior and posterior approaches are effective in improving neurological recovery after CSCI, the optimal surgical approach for treating CSCI remains a subject of ongoing debate. While anterior approaches are generally favored for ventral spinal cord decompression due to disc protrusion or vertebral body fragments (17, 18), posterior approaches are considered for facet dislocations unamenable to closed reduction, barring concurrent disc herniation (9). Our meta-analysis, regardless of original pathology, found no significant difference in neurofunctional recovery, as measured by ASIA, JOA scores, and Recovery Rate (RR), between the anterior and posterior approaches. These findings align with those of Brodke et al. (9), where 52 patients with CSCI were randomized to either anterior or posterior stabilization and fusion. The authors concluded that there is no clear evidence favoring either the anterior or posterior approach in treating persons with cervical spinal cord injuries.

Both anterior and posterior surgical approaches to CSCI are associated with distinct risks and complications. The anterior approach is linked with postoperative issues such as odynophagia, hoarseness, and persistent neck discomfort, potentially resulting from the surgical method or chronic irritation due to implants (19, 20). Conversely, the posterior approach may result in a heightened risk of neck pain and wound infection, though instances of odynophagia are comparatively rare. Our study discerned no significant difference in complication rates between the two surgical methods. This equivalence was also observed in our comparative analysis of operation duration and intraoperative hemorrhage.

These outcomes challenge the preconceived notion that the anterior approach may be superior to the posterior. Variability in surgical techniques—such as ACCF and ACDF for anterior surgeries, and laminectomy and laminoplasty for posterior procedures—may account for this finding (21, 22). Supportive of our results is a prospective multicenter study that found both anterior and posterior interventions to be equally efficacious in enhancing neurological outcomes in persons with cervical spondylotic myelopathy (CSM). Moreover, several systematic reviews concur that despite the anterior approach potentially facilitating better immediate postoperative neurological function, no significant differences are observed in long-term neurological recovery rates between the two strategies (23–25). Nonetheless, the literature presents a dichotomy of perspectives. Certain authors advocate for the anterior approach, citing significant improvements in JOA scores and neurological restoration compared to the posterior method (26). Conversely, retrospective analyses suggest the posterior approach may reduce the risk of pneumonia, sepsis, surgery-related complications, and mortality within a year of the procedure (27). Furthermore, in a meta-analysis, Zhu, B. et al. identified a significantly higher rate of complications associated with anterior surgeries (23).

The lack of consensus on the superiority of anterior vs. posterior approaches in managing CSCI underscores the complexity of surgical decision-making. Influential factors include the etiology of the condition, the extent of involvement, and, critically, the surgeon's expertise. With this regard, a workflow based on a new classification proposed by the AOSpine Spinal Cord Injury Knowledge Forum could be followed (28). It emphasizes spinal stability, cord compression and neurological status using ASIA system as three major considerations. But the choice between surgical strategies remains a nuanced decision, necessitating a personalized approach based on a thorough evaluation of individual factors. Therefore, it is highly recommended that both approaches should be included as a fundamental component of surgical training for each spine surgeons (29).

## Limitations and conclusions

This meta-analysis has several limitations, including the predominance of non-randomized control trials, a general comparison without accounting for specific surgical techniques, and varying follow-up durations across studies. Also, the impacts of on-site first aid management and postsurgical physical excersices were not taken into analysis due to the limited data availability. However, it is suggested that the allocation of neurotrauma service resource as well as the neurorehabilitation may affect the outcome in both traumatic brain and spinal cord injury patients, which are encouraged to be enrolled in future studies (30). Despite these limitations, our study provides valuable insights into the surgical outcomes and risks associated with the anterior and posterior approaches for CSCI. Surgeons should be well-versed in the merits and drawbacks of each approach to make informed decisions in consultation with their individuals.

## Data Availability

The original contributions presented in the study are included in the article/Supplementary Material, further inquiries can be directed to the corresponding author.
